# Genome-Wide Identification and Gene Expression Analysis of the OTU DUB Family in *Oryza sativa*

**DOI:** 10.3390/v14020392

**Published:** 2022-02-14

**Authors:** Qiannan Liu, Tingyun Yan, Xiaoxiang Tan, Zhongyan Wei, Yanjun Li, Zongtao Sun, Hehong Zhang, Jianping Chen

**Affiliations:** 1 College of Plant Protection, Northwest Agriculture and Forestry University, Yangling, Xianyang 712100, China; liu18821690406@163.com (Q.L.); tingyunyan@163.com (T.Y.); txx558@126.com (X.T.); 2 State Key Laboratory for Managing Biotic and Chemical Threats to the Quality and Safety of Agro-Products, Key Laboratory of Biotechnology in Plant Protection of Ministry of Agriculture and Zhejiang Province, Institute of Plant Virology, Ningbo University, Ningbo 315211, China; weizhongyan@nbu.edu.cn (Z.W.); liyanjun@nbu.edu.cn (Y.L.); sunzongtao@nbu.edu.cn (Z.S.)

**Keywords:** rice, OTU DUB, genome-wide, plant hormone treatment, virus infection

## Abstract

Ovarian tumor domain (OTU)-containing deubiquitinating enzymes (DUBs) are an essential DUB to maintain protein stability in plants and play important roles in plant growth development and stress response. However, there is little genome-wide identification and analysis of the *OTU* gene family in rice. In this study, we identified 20 genes of the OTU family in rice genome, which were classified into four groups based on the phylogenetic analysis. Their gene structures, conserved motifs and domains, chromosomal distribution, and *cis* elements in promoters were further studied. In addition, *OTU* gene expression patterns in response to plant hormone treatments, including SA, MeJA, NAA, BL, and ABA, were investigated by RT-qPCR analysis. The results showed that the expression profile of *OsOTU* genes exhibited plant hormone-specific expression. Expression levels of most of the rice *OTU* genes were significantly changed in response to rice stripe virus (RSV), rice black-streaked dwarf virus (RBSDV), Southern rice black-streaked dwarf virus (SRBSDV), and Rice stripe mosaic virus (RSMV). These results suggest that the rice *OTU* genes are involved in diverse hormone signaling pathways and in varied responses to virus infection, providing new insights for further functional study of *OsOTU* genes.

## 1. Introduction

Ubiquitin was discovered in 1975 [1]. Ubiquitination is involved in regulating biochemical processes in many eukaryotic organisms and is an important basis for protein stability and function. Ubiquitination is accomplished by E1 activation, E2 conjugation, and E3 enzyme ligation, resulting in ubiquitination, which ultimately is covalently linked to the lysine residue of the substrate. Ubiquitin can be covalently bound to the lysine residue of the ubiquitin. The presence of seven different types of lysine residues eventually form unique forms of polyubiquitin chains [2,3].

The modification of cellular proteins by ubiquitin (Ub) is a reversible process. Like ubiquitination, deubiquitination is a highly regulated process that is involved in many cellular functions, including cell cycle regulation [4], proteasome- and lysosome-dependent protein degradation [5,6], gene expression [7], DNA repair [8], kinase activation [9], and microbial pathogenicity [10]. The reversible reaction of ubiquitination is regulated by about 100 deubiquitinases (DUBs), among which DUBs containing ovarian tumor domain (OTU) are common and specifically cleave different types of ubiquitin chains on substrate proteins [11]. The OTU protein family is a highly specific ubiquitin isopeptidase that can remove ubiquitin from proteins, and is conserved from plants to humans. Otubain retains some conserved domains in evolution, including the OTU domain with an active cysteine protease triad, an Ub interaction motif (UIM)-like motif phi-xx-A-xxxs-xxx-Ac (where phi represents aromatic amino acids, x represents any amino acids, Ac represents acidic amino acids), a nuclear localization signal, and Ub-associated (UBA) domain [12].

There were many studies on OTU family proteins, especially in humans and *Arabidopsis*. Most of the 16 human OTU DUBs are associated with cell signaling cascades [13]. OTUB1 (ubiquitin aldehyde binding protein 1 containing OTU domain) shows preference for K48-linked strand cleavage [14,15] and regulates the synthesis of K63-Ub in an irregular manner. The amino terminal residues of the OTU domain inhibit the synthesis of K63-Ub by binding to an E2 ubiquitin ligase UBC13 [16]. *Arabidopsis* OTU DUBs are also a kind of cysteine protease, which specifically recognize and cleave three common types of ubiquitin linkages, including M1, K48, and K63 [17]. Bacterial and viral pathogens have a new evolutionary strategy that manipulate host ubiquitin signaling by utilizing the OTU fold to destroy host signaling [18]. CCHFV OTU and the N-terminal of RSV-encoded Pc1 protein contain an ovarian tumor (OTU) protease domain, which cleaves K48-linked and K63-linked polyUb chains [19]. Furthermore, the activity is abolished when the corresponding putative catalytic triads Asp, Cys, and His [20] are mutated, which may present a similar protein structure and function to escape ubiquitin-dependent antiviral responses [21]. Obviously, OTU plays an important role in plant immunity and pathogen invasion by regulating the ubiquitination of organisms and cleaving specific ubiquitin chains.

The *OTU* family genes in several plant species have been comprehensively analyzed. However, little information about the OTU family was available in rice now. Rice belongs to the *Poaceae* family and is an important cereal crop that supports the global food supply [22]. However, the yield of rice is affected by many factors, including biotic stress and abiotic stress. Phytohormones are very important signals in plants; they play crucial roles in plant development and in response to stresses caused by biotic agents or abiotic factors. Pathogen invasion is a kind of biological stress. In the arms race between pathogens and plants, pathogens gradually evolved a new mechanism. They may synthesize cysteine protease with OTU domains themselves or interact with OTUs in plants to regulate ubiquitination and deubiquitination in plants, further affecting plant immune responses. In this study, we firstly investigated the genome-wide analysis of the rice *OTU* gene family and studied the expression pattern of *OsOTU* genes in response to different hormone treatments and various virus infection.

## 2. Materials and Methods

### 2.1. Genome Identification of OTU Family Genes in Rice, Arabidopsis, and Maize

The whole genome sequences of rice, *Arabidopsis*, and maize were extracted from Phytozome’s Osativa Genome 7.0 version, TAIR, and ensembled plant Zea_mays.B73_RefGen_v4.pep version [23]. According to previous studies on *Arabidopsis* OTU family proteins, protein sequences of *Arabidopsis* OTU family were downloaded [17]. OTU was used as a keyword to search and download protein sequences, while *OsOTU2* and *Zm00014a_002048* were used as protein queries for BLASTp searches to find OTU family members in rice and maize. With a cut-off of Evalue <10^−10^, a distance score value of ≥100 was used to screen out candidate genes. Further screening obtained 20 rice OTUs and 15 maize OTUs. The theoretical isoelectric point (pI) of them was then calculated by ExPAsy [24,25]. 

### 2.2. Multiple Sequence Alignment and Phylogenetic Analysis

The amino acid sequence encoding CDS of OTU proteins from rice, *Arabidopsis*, and maize was downloaded for phylogenetic analysis. ClustalW was used to align all acquired sequences [15]. MEGA6.0 software was used to construct a phylogenetic tree with 1000 bootstrap tests based on neighbor-joining (NJ) methods [14,16].

### 2.3. Analysis of Conserved Gene Domains and Motifs

Pfam website (http://pfam.xfam.org/ (accessed on: 18 September 2021)) was used to obtain rice gene domains data and TBtools was used for visual analysis [26]. Gene motif data for rice *OTU* genes were obtained from the MEME website (https://meme-suite.org/meme/doc/meme.html (accessed on: 18 September 2021)) and mapped with TBtools Visualize Domain Pattern methods [27,28]. Their conserved motifs were predicted.

### 2.4. Gene Structure and Chromosomal Distribution of rice OTU Gene Analysis

The genome annotation file (protein) for *Osativa* was acquired from phytozome [29]. All the results were rearranged by TBtools Visualize Gene Structure. The chromosome distribution information of the target gene was extracted by TBtools Gene Location Visualize methods, according to the description of the distribution of rice genes on chromosomes in the gene annotation file of *Osativa*.

### 2.5. Prediction Cis-Acting Elements of Rice OTU Genes

The target gene CDS upstream 2000 bp sequence from rice whole genome sequence was extracted with TBtools. PlantCARE (http://bioinformatics.psb.ugent.be/webtools/plantcare/html/ (accessed on: 20 September 2021)) is a database of plant *cis*-acting regulatory elements, enhancers, and repressors [30,31]. The conserved *cis*-acting elements in the promoter region of the target gene were predicted by the PlantCARE Database. All results were filtered manually and presented with TBtools.

### 2.6. Plant Materials Acquisition and Hormone Treatments

The seeds of Nipponbare (NIP) rice were grown in greenhouse at 30 °C, treated with 14 h light and 10 h darkness for 14 days. The mother liquor was prepared with absolute ethanol as the solvent and diluted with sterile distilled water containing 0.1% Triton X-100 to 500 μM of SA, 100 μM of MeJA, 5 μM of NAA, 10 μM of BL, and 50 μM of ABA, respectively. The same volume of sterile distilled water with 0.1% Triton X-100 was used as a mock control. The 14-day-old Nipponbare (NIP) rice seedlings were sprayed with the specified concentration of the hormone, and the control was treated simultaneously as the same. Samples were collected at 3 h, 6 h, and 12 h after spraying, and stored at −80 °C until the total RNA was extracted.

### 2.7. Rice Viruses Infect Plant Materials

The methods of rice virus inoculation were conducted following our described previously [32,33]. The 10-day-old NIP, ZH11, ZS97, and ZH5 seedlings were inoculated with SBPH carrying RSV, SBPH carrying RB, WBPH carrying SRBSDV, and Leaf hopper carrying RSMV, respectively. Approximately 20 seedlings per treatment and 2 insects per seedling were fed for 3 days. After then, the insects were removed and grown in greenhouse to observe the symptoms. Three biological replicates were conducted for each treatment.

### 2.8. RNA Extraction and RT-qPCR

After inoculation, the seedlings of NIP, ZH11, ZS97, and ZH5 were collected at 20 to 30 dpi, and the samples were ground individually in liquid nitrogen. TRIzol reagent was used to extract the samples in cold conditions (Invitrogen, Carlsbad, CA, USA), according to the manufacturer’s protocol. The reverse transcription of total RNA was used to obtain cDNA with the Tiangen Rapid Quantitative RT Kit with gDNase (Tiangen, Beijing, China) [34]. RT-qPCR was used to analyze the gene expression level by qPCR SYBR green master mix on a real-time PCR machine (ABI) [35]. *OsUBQ5* (AK061988) was used as an internal control [36]. The data were calculated by the 2^−ΔΔCT^ method [37]. Three biological replicates were conducted for each experiment. The primers used for RT-qPCR are listed in Table S1.

## 3. Results

### 3.1. Identification and Analysis of OTU Family in Rice

In this study, we performed a genome-wide analysis to identify members of the OTU family in the rice genome. We first used the known OTU sequences in *Arabidopsis* and rice to perform BlastP search sequences in the rice genome and rice full-length cDNA databases. By NCBI CD search and comparison with known rice OTU family proteins, we finally identified 20 non-redundant rice *OTU* genes, including 14 known rice *OTU* genes, and named them *OsOTU1* to *OsOTU20*. All identified rice OTU protein gene IDs, CDS lengths, positions of conserved domains, and isoelectric points are listed in Table 1. In addition, the same method was used to identify 15 non-redundant maize *OTU* genes to compare the OTU family proteins between monocots (Table S2).

### 3.2. Analysis of Domain Organization, Conserved Motifs, and Phylogenetics of OTU

**Figure 1:**
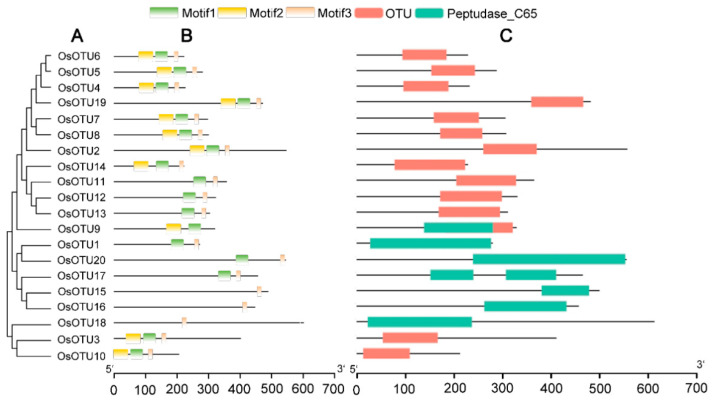
Phylogenetic tree, motif analysis of OTUs, and conserved domain. (**A**) Phylogenetic analysis of OsOTU proteins in rice. (**B**) Conserved motif analysis of OsOTUs using MEME tools. Motifs are shown in different colored rectangles. (**C**) Conserved domain distributions of the out proteins in rice. The OTU domain and The Peptudase C65 domain are indicated in red and blue rectangles, respectively.

**Figure 2:**
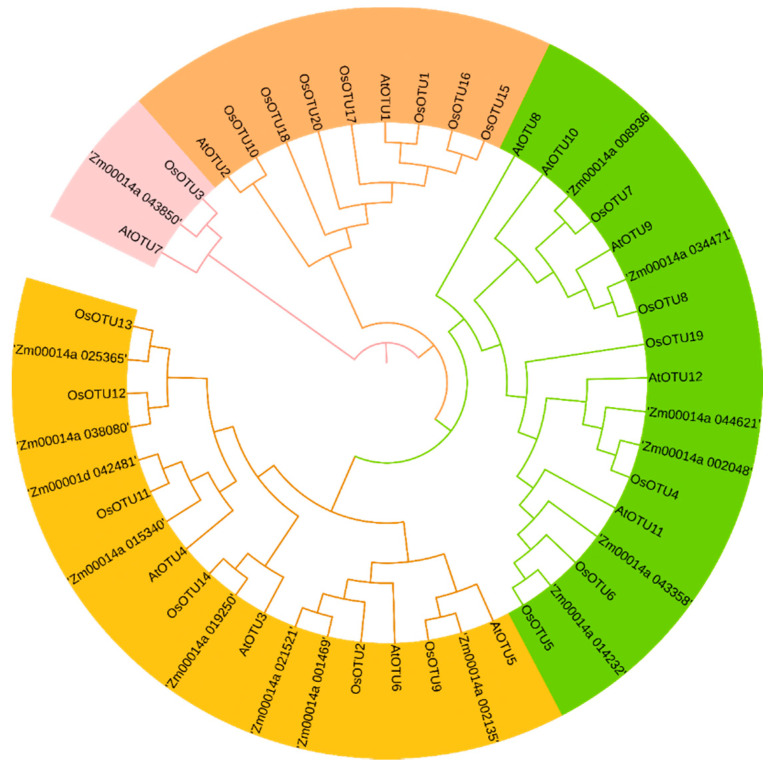
Phylogenetic analysis of OsOTU family proteins from *Arabidopsis thaliana*, *Oryza sativa*, and *Zea mays*. The phylogenetic tree was constructed using MEGA6.0 software by the neighbor-joining method with 1000 bootstrap replicates.

Among the 20 rice *OTU* genes, *OsOTU4*, *OsOTU5*, *OsOTU6*, *OsOTU7*, *OSOTU8*, and *OSOTU19* belonged to the same group with four *Arabidopsis OTU* genes *AtOTU8-12*, which only contain a C-terminal OTU domain (Figure 1 and Figure 2). *OTU* genes are divided into four groups by the phylogenetic analysis of identified rice, *Arabidopsis*, and maize *OTU* gene family (Figure 2). On the other hand, all maize *OTU* genes only contain the OTU domain. It was found that some rice *OTU* family genes contain another domain, the peptidase C65 domain. Moreover, the *Arabidopsis* *AtOTU1*, which also contains the peptidase C65 domain, is grouped together. Cysteine proteases, known as peptidase C65, were involved in the precise cleavage at the specific deubiquitylation at the Ub–protein bond. It is reported that Otubain carries several key conserved domains [12]. To identify common motifs among the different groups of *OTU* genes, we used the MEME motif search tool and mapped the results with TBtools. Three conserved motifs were identified (Appendix A). OTUs of the same group exhibited similar motif distribution patterns.

### 3.3. Gene Structure and Chromosomal Distribution Analysis of Rice OTU Genes

**Figure 3:**
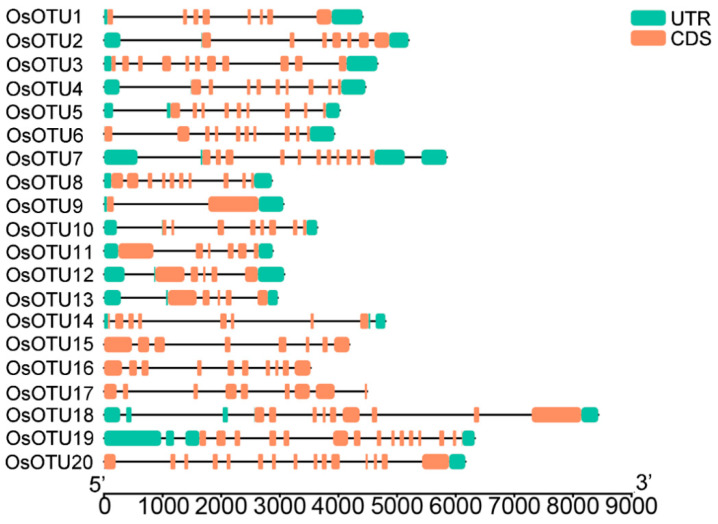
Exon–intron structure analysis of OsOTU family proteins. Green rectangles indicate untranslated regions (UTRs) from 5′and 3’, red rectangles indicate exons, and black lines indicate introns.

The diversity of the exon–intron structure in the gene family provides an important basis for analyzing the evolution and function of gene family members [38]. We analyzed the structural diversity of rice *OTU* genes, as shown in Figure 3, which displays the exon–intron structure. The 20 genes contain both introns and exons, and the exons are not contiguous. The existence of introns made eukaryotic genes become discontinuous genes or break genes. They all have at least two exons and one intron. Mature mRNA production requires intron splicing from primary transcripts [39]. Since they all have multiple exons and introns, there may be multiple splicing methods. It also means that multiple mature RNAs may be produced through alternative splicing. We searched the Rice Genome Annotation Project database to further verify this hypothesis, and found that these *OTU* family genes have two or more transcripts. Of particular significance, *OsOTU10* even has 10 different transcripts. The chromosome position analysis of the rice *OTU* family genes in 12 rice genome chromosomes is shown in Figure S1.

### 3.4. Prediction of OsOTU Cis-Acting Elements

**Figure 4:**
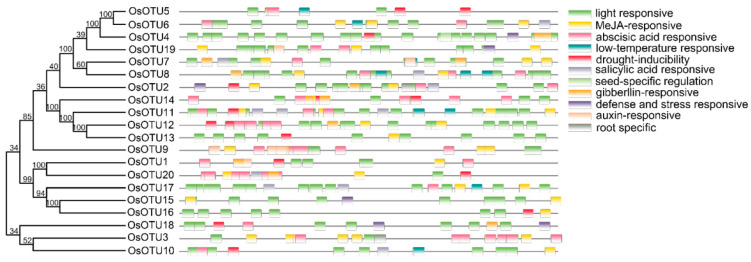
Prediction and analysis of *OsOTU* gene *cis*-acting elements. The potential *cis*-regulatory elements in the promoter regions 2000 bp upstream of the *Oryza sativa* were predicted by the PlantCARE website. Different colors indicated the elements related to growth and development (seed-specific regulation and root-specific), plant hormones (methyl jasmonate, abscisic acid, salicylic acid, gibberllin acid and auxin), and stress responsiveness (light, low temperature, and drought inducibility, defense and stress responsive).

Large-scale prediction of promoter sequences and their contributing *cis*-acting elements has become routine due to recent advances in transcriptomic technologies and genome sequencing of several plants [40]. Promoter analysis is of great significance in studying the regulation of natural genes and developing available transgenic crops [41]. To reveal the roles of *cis*-regulatory elements of rice *OTU* genes under biotic and abiotic stress responses, we analyzed the promoter regions (2000 bp upstream of the translation start site) of the 20 rice *OTU* genes using the Plant CARE Database. We identified these *cis*-regulatory elements and further visualized them with TBtools (Figure 4). They are divided into seven functional groups: light response, promoters, binding sites, development, oxygen, hormone, and adversity stress. The hormone response is particularly rich. Five hormone responses, including abscisic acid (ABA), gibberellin (GA), methyl jasmonate (MeJA), salicylic acid (SA), and auxin, were identified (Figure 4). Almost all rice *OTU* genes contain *cis*-acting regulatory elements related to hormone regulation, indicating that the rice *OTU* family genes are subject to hormonal factors. In addition, some are related to tissue-specific expression regulation. By further analysis on the phylogenetic tree, we found that the closely related OTU proteins (such as OTU5 and OTU6, OTU12 and OTU13, OTU1 and OTU20, and OTU15 and OTU16) have distinct predicted regulatory elements in their promoters (Figure 4). We speculate that this may be due to the functional diversity of *OTU* family genes and the different expression patterns. 

### 3.5. Expression Patterns of Rice OTU Genes under Hormone Treatments

**Figure 5:**
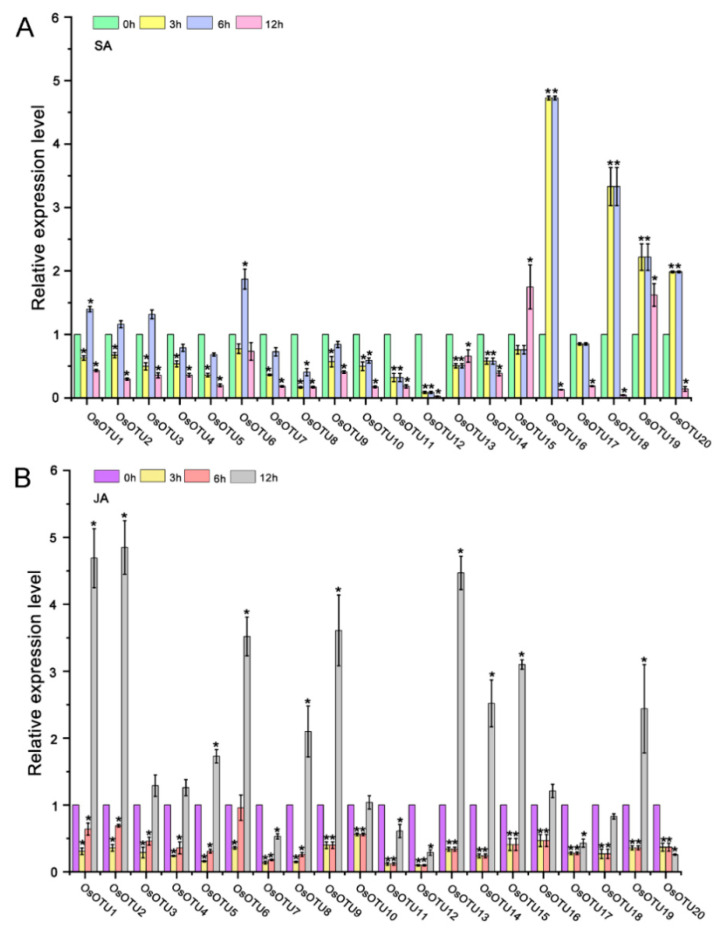
The expression pattern of *OsOTU* genes under different hormone treatments. Plants were treated with SA (500 μM) (**A**), MeJA (100 μM) (**B**). Samples were collected at 0, 3, 6, and 12 h. The *OsUBQ5* gene was the reference gene used to calibrate the relative gene expression. Error bars represent ±SE (*n* = 3). One-way analysis of variance was conducted by Duncan’s new multiple-range test, *n* = 3. * indicates significant differences from the mock control at *p* ≤ 0.05 by Fisher’s least significant difference tests.

**Figure 6:**
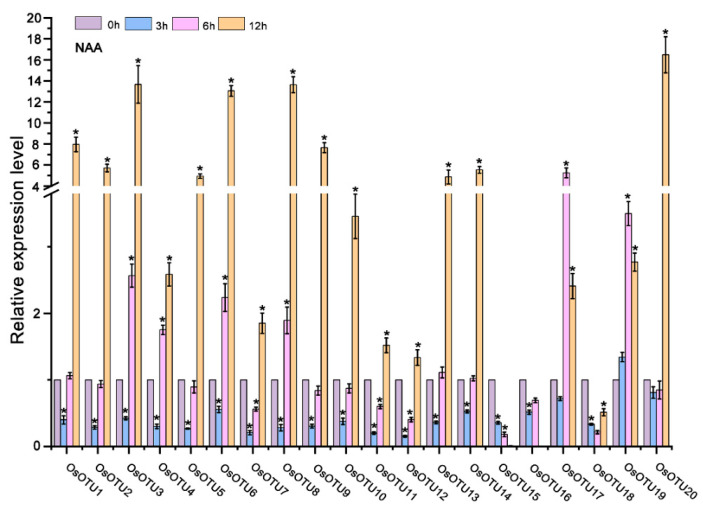
The expression pattern of *OsOTU* genes under different hormone treatments. Plants were treated with NAA (50 μM). Samples were collected at 0, 3, 6, and 12 h. The *OsUBQ5* gene was the reference gene used to calibrate the relative gene expression. Error bars represent ±SE (*n* = 3). One-way analysis of variance was conducted by Duncan’s new multiple-range test, *n* = 3. * indicates significant differences from the mock control at *p* ≤ 0.05 by Fisher’s least significant difference tests.

**Figure 7:**
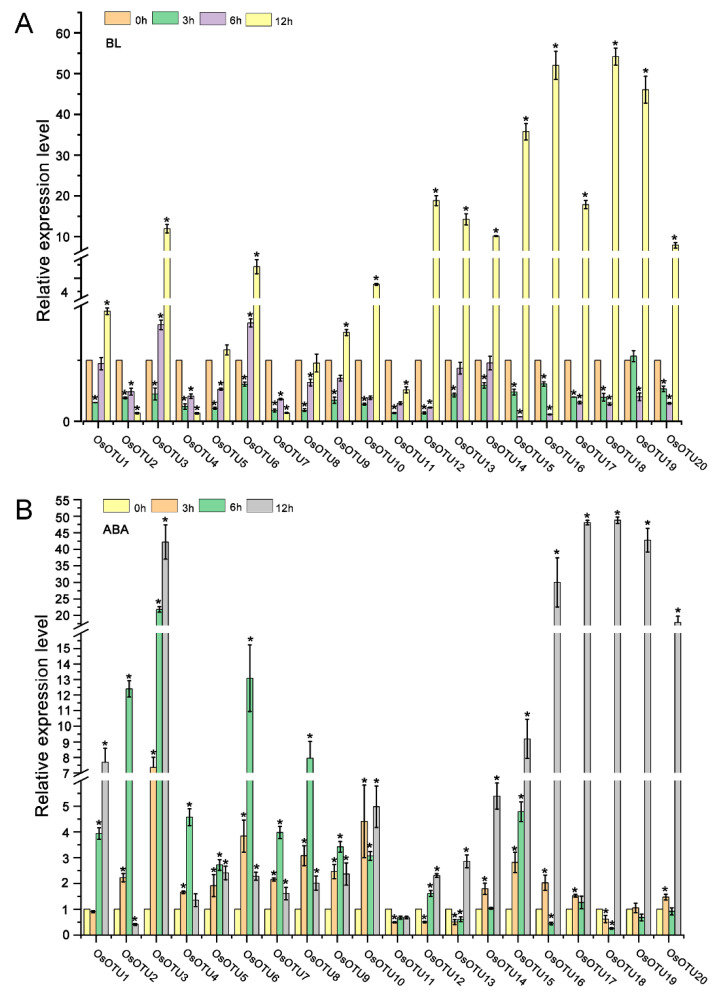
The expression pattern of *OsOTU* genes under different hormone treatments. Plants were treated with BL (10 μM) (**A**), ABA (50 μM) (**B**). Samples were collected at 0, 3, 6, and 12 h. The *OsUBQ5* gene was the reference gene used to calibrate the relative gene expression. Error bars represent ±SE (*n* = 3). One-way analysis of variance was conducted by Duncan’s new multiple-range test, *n* = 3. * indicates significant differences from the mock control at *p* ≤ 0.05 by Fisher’s least significant difference tests.

Our analysis of cis-acting elements in the promoters of *OTU* family genes suggest that their expression might be regulated by plant hormones. To verify this hypothesis, we analyzed the expression patterns of rice *OTU* genes under different hormone treatment, including SA, MeJA, 1-naphthylacetic acid (NAA), epibrassinolide (BL), and ABA by RT-qPCR. We found that almost most of the rice *OTU* genes were upregulated by four hormones, except SA, at 12 h after treatment. Interestingly, most of rice *OTU* genes had a peak in the first 6 h after SA treatment and dropped sharply after 12 h, except *OsOTU13, OsOTU15*, and *OsOTU19* (Figure 5, Figure 6 and Figure 7). Moreover, half of them were downregulated after SA treatment. *OTU* genes are more responsive to BL and ABA compared to JA and NAA. This was obvious in *OsOTU16*, *OsOTU17*, *OsOTU18*, and *OsOTU19*. However, *OsOTU2*, *OsOTU4*, *OsOTU7*, and *OsOTU11* were downregulated after BL treatment. In addition, all 20 rice *OTU* genes were steadily upregulated until they reached a peak at 12 h under MeJA treatment. Differently, 19 of the 20 rice *OTU* genes were downregulated first and then upregulated under SA treatment. Together, most of the rice *OTU* genes were upregulated under MeJA, NAA, BL, and ABA treatment, except SA (Figure 5, Figure 6 and Figure 7). Half of the rice *OTU* genes were downregulated after SA treatment. Through further analysis on the relationship between the prediction of the hormone response elements in the *cis*-acting elements of OTU family promoters (Figure 4) and the *OTU* genes in response to hormone (Figure 5, Figure 6 and Figure 7), we found that *OsOTU11* and *OsOTU17* were enriched in SA and JA response elements, but their expression levels were significantly inhibited after SA and JA treatment. We speculate that SA and JA may negatively regulate the expression of *OsOTU11* and *OsOTU17*. At the same time, we found that *OsOTU15*, *OsOTU16*, and *OsOTU18* do not have an auxin response element and that their expression levels were significantly inhibited after NAA treatment. Most of the *OTU* genes were enriched in ABA response elements and upregulated after ABA treatment (Figure 5, Figure 6 and Figure 7). Our data revealed that most rice *OTU* genes responded to hormones specifically with different expression patterns.

### 3.6. Analysis of Rice OTU Genes Expression Profiling after RSV, RBSDV, SRBSDV, and RSMV Infection

Rice stripe virus (RSV) is an important pathogen of rice and is mainly transmitted by the insect vector small brown planthopper (SBPH), *Laodelphax striatellus* [42]. Rice stripe disease is caused by RSV results in severe yield losses in rice production [43,44,45]. Rice black-streaked dwarf virus (RBSDV) belongs to the *Fijivirus* genus [46], transmitted by SBPH. It caused rice black-streaked dwarf disease and maize rough dwarf disease [47,48]. Another member of the *Fijivirus* genus is the Southern rice black-streaked dwarf virus (SRBSDV) which is transmitted by the rice white backed planthopper (WBPH), *Sogatella furcifera* [49,50]. Rice stripe mosaic virus (RSMV), a new *cytorhabdovirus* (family *Rhabdoviridae*), is transmitted by the leafhopper (*Recilia dorsalis*) in a persistent-propagative manner [51].

**Figure 8:**
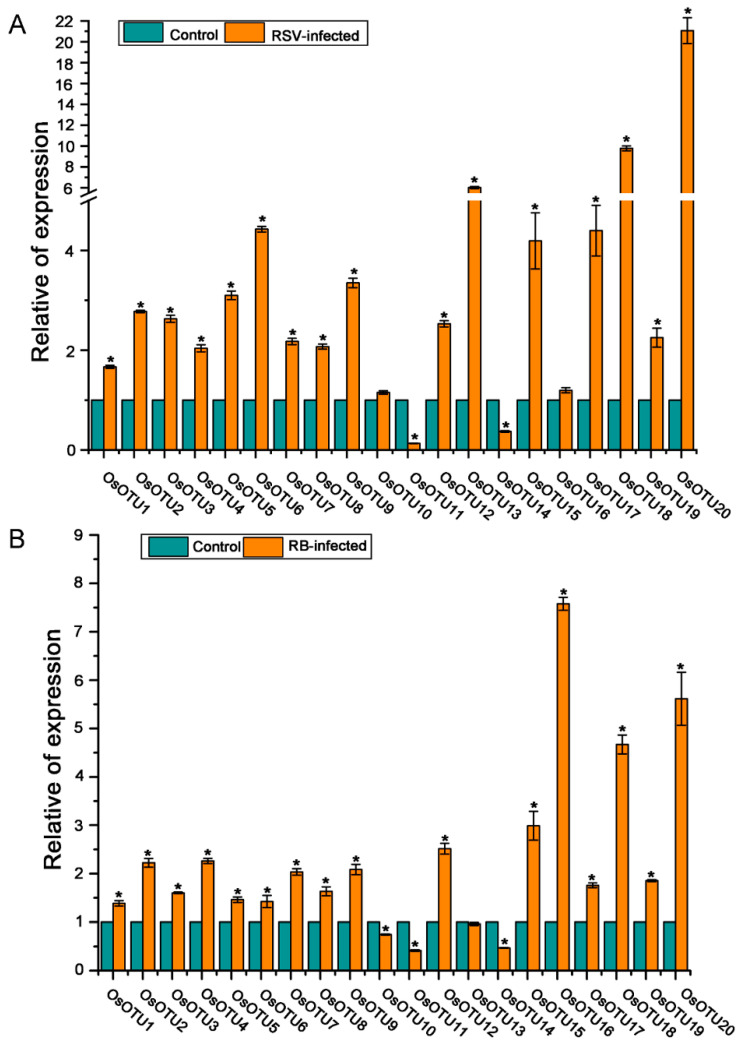
The expression of *OsOTU* genes in response to RSV (**A**) and RBSDV (**B**) infection. Three biological replicate experiments were performed for each treatment and gene expression was detected by RT-qPCR. Samples were collected at 25 days post-inoculation (dpi) from RSV-infected plants and at 45 dpi from RBSDV-infected plants. The *OsUBQ5* gene was used to normalize the relative gene expression. Error bars represent ±SE (*n* = 3). * indicates significant differences from the mock control at *p* ≤ 0.05 by Fisher’s least significant difference tests.

In order to further investigate whether rice *OTU* genes participate in virus infection, the expression levels of rice *OTU* genes were analyzed in RSV, RBSDV, SRBSDV, and RSMV-infected rice. RT-qPCR assays showed that the expression levels of most rice *OTU* genes, except *OsOTU11* and *OsOTU14*, were dramatically raised in RSV-infected rice plants compared to mock control plants at 20 days post-inoculation (dpi). In addition, *OsOTU10* was not affected by RSV infection (Figure 8A). Similarly, the expression levels of most rice *OTU* genes were upregulated in RBSDV-infected rice plants compared to mock control plants at 25 dpi, except *OsOTU10*, *OsOTU11*, and *OsOTU14* (Figure 8B).

**Figure 9:**
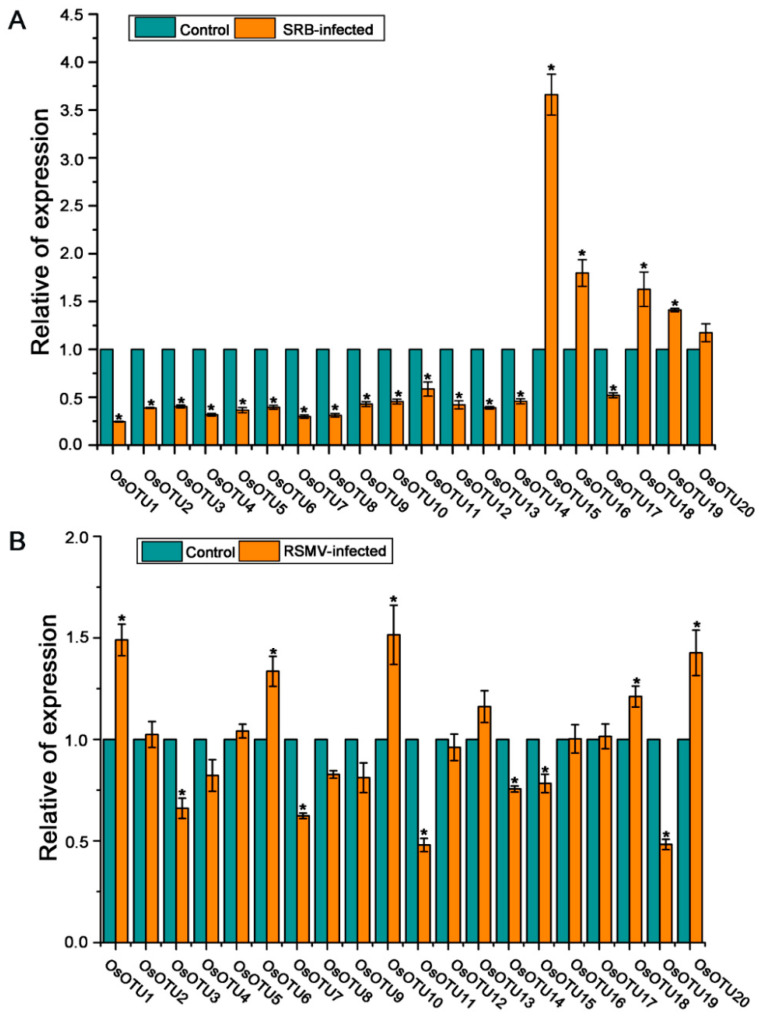
The expression of *OsOTU* genes in response to SRBSDV (**A**) and RSMV (**B**) infection. Three biological replicate experiments were performed for each treatment and gene expression was detected by RT-qPCR. Samples were collected at 40 dpi from RSV-infected plants and at 45 dpi from RSMV-infected plants. The *OsUBQ5* gene was used to normalize the relative gene expression. Error bars represent ±SE (*n* = 3). * indicates significant differences from the mock control at *p* ≤ 0.05 by Fisher’s least significant difference tests.

In contrast, most rice *OTU* genes, including *OsOTU10*, *OsOTU11*, and *OsOTU14*, were significantly downregulated under SRBSDV virus infection at 30 dpi. Differently, *OsOTU15*, *OsOTU16*, *OsOTU18*, and *OsOTU19* were increased (Figure 9A). We then investigated the expression levels of rice *OTU* genes in RSMV-infected rice. Interestingly, almost half of *OTU* genes were unaffected by RSMV infections at 30 dpi. Half of the remaining genes are upregulated and the others are downregulated (Figure 9B).

We found that the expression levels of *OsOTU18* and *OsOTU20* were all upregulated to RSV, RBSDV, SRBSDV, and RSMV infection, while their expression levels were significantly downregulated after JA treatment, indicating that they may be susceptible to these four viruses. Our results showed that most rice *OTU* genes respond to viral infection. Additionally, the expression levels of these genes are different to different virus infection. The results indicated that the same gene has different expression levels under different virus infections.

**Figure 10:**
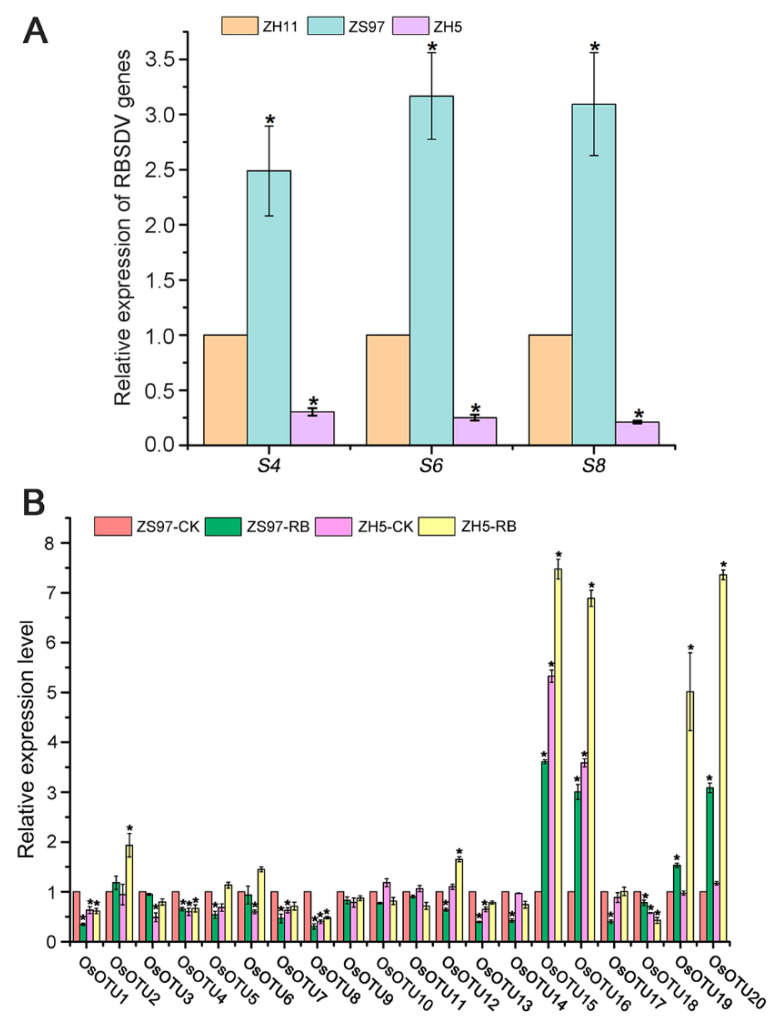
Differential expression analysis of resistant ZH5 and susceptible ZS97 plants after RBSDV infection. (**A**) RT-qPCR results showing the relative expression of viral RNA (RBSDV genomic RNA segments S2, S4, and S6) in RBSDV-infected ZH11, ZS97, and ZH5 plants. (**B**) RT-qPCR verification of the expression of *OsOTU* genes between resistant ZH5 and susceptible ZS97 after RBSDV infection. Three biological replicate experiments were performed. Samples were collected at 30 dpi from RBSDV-infected ZH5 and ZS97 plants. The *OsUBQ5* gene was used to normalize the relative gene expression level. Error bars represent ±SE (*n* = 3). * indicates significant differences from the mock control at *p* ≤ 0.05 by the Fisher’s least significant difference tests.

Our preliminary studies showed that the rice variety ZH5 displayed high resistance to RBSDV infection, while ZS97 exhibited susceptibility. The RT-qPCR assay showed that the expression levels of RBSDV *S4, S6*, and *S10* RNA in ZS97 were higher compared with those in Zhonghua 11 (ZH11) control plants, while the expression levels were significantly reduced in ZH5 plants (Figure 10A). Relevant primers are derived from [21]. The results suggested ZH5 that indeed displayed high resistance, while ZS97 exhibited susceptibility to RBSDV infection. To identify the candidate *OsOTU* genes associated with virus-resistant studies in rice, we performed differential expression analysis of resistant ZH5 and susceptible ZS97 after RBSDV infection. After 30 dpi, the seedlings of ZH5 and ZS97 were collected, and the samples were ground individually in liquid nitrogen. RT-qPCR assays showed that more than half of the expression levels of *OsOTU* genes were significantly downregulated, except *OsOTU15, OsOTU16*, *OsOTU19*, and *OsOTU20* which were dramatically upregulated in susceptible variety ZS97 plants after RBSDV infection (Figure 10B). In the resistance variety of ZH5, one-third of *OTU* genes, including *OsOTU2, OsOTU12, OsOTU15, OsOTU16, OsOTU19*, and *OsOTU20,* were significantly increased under RBSDV infection (Figure 10B). Interestingly, we found that *OsOTU15* and *OsOTU16* showed differential expression patterns between ZH5 and ZS97, with a high expression level in ZH5 and a low expression level in ZS97 plants (Figure 10B). The results display that these two genes can play an important role in the process of viral infection. At present, although the transient expression of *OTU* genes can be performed in the model plant, it was not easy for rice viruses to infect *Arabidopsis* and *N. benthamiana* by mechanical inoculation [52]. Recently, a series of studies have published on the tolerance of genes to RBSDV infection, all of which are achieved by constructing transgenic rice and then inoculate RBSDV [32,33,53,54]. In the future, we will further verify the resistance of OsOTU15 and OsOTU16 to rice black-streaked dwarf virus disease (RBSDVD) by constructing transgenic plants in the future.

## 4. Discussion

As reported in several published studies, *OTU* family genes regulate ubiquitination pathways by specifically cleaving different types of ubiquitin chains, and play an important role in protein stability and stress response in plants [17,55]. However, there are few related studies in the structure and regulatory functions of *OTU* family genes in rice. In this study, we systematically analyzed the phylogeny, conserved domains, gene structure, chromosome distribution, and *cis*-acting elements of rice *OTU* deubiquitinase. In addition, the expression patterns of *OTU* family genes under five different hormone (SA, JA, NAA, BL, and ABA) treatments and four different rice virus (RSV, SRBSDV, RBSDV, and RSMV) infections were investigated.

Twenty *OTU* family genes were identified from rice, fifteen from maize, and twelve from *Arabidopsis*. The genome-wide analysis of rice *OTU* family genes show that they were phylogenetically clustered into four groups. *OTU* family genes have two conserved domains. Most genes that only contain OTU domains are grouped with *Arabidopsis* genes that only contain OTU domains. The other domain, the peptidase C65 domain, is grouped with *AtOTU1*, i.e., the only member of *Arabidopsis* that contains the peptidase C65 domain. Cysteine proteases, known as peptidase C65, involved in precise cleavage at the specific deubiquitylation at the Ub–protein bond. Thus, the peptidase C65 domain is as important as the OTU domain. We discovered that all rice *OTU* family genes are discontinuous genes unexpectedly. They all have multiple exons and introns, which means that there may be multiple splicing methods. This may just explain why there are multiple transcripts of rice *OTU* family genes, but this needs further study.

We found that almost all rice *OTU* genes contain *cis*-acting regulatory elements related to hormone regulation. In further studies, we demonstrated that rice *OTU* genes were mostly downregulated by SA and upregulated by the other four hormones at 12 h after treatment. The expression patterns of the *OTU* gene family were similar by MeJA and NAA treatment in rice, but different in BL and ABA. The results indicated that the *OTU* gene family are obviously involved in hormone response and exhibit different expression patterns. By analyzing the response of *OTU* family genes to plant hormones, we speculated that SA and JA may negatively regulate the expression of *OsOTU11* and *OsOTU17*. Most *OTU* genes enriched in ABA response elements are upregulated after ABA treatment. We tested the expression patterns of rice *OTU* family genes under the infection of different plant RNA viruses. We conclude that most rice *OTU* genes were dramatically upregulated after RSV and RBSDV infection except *OsOTU11* and *OsOTU14*. In contrast, most rice *OTU* genes, including *OsOTU11* and *OsOTU14*, were significantly downregulated under SRBSDV. Even half of *OTU* genes were unaffected by RSMV infections. Rice *OTU* family genes play an important role in the process of virus invasion and respond to the invasion of different viruses in different ways. The participation of genes in these processes will require further investigation.

It has been reported that SA is essential for plants to viruses and pathogens in the basic resistance. Exogenous spraying of SA can significantly increase the resistance of rice to RBSDV [56]. We found that half of the rice *OTU* genes were downregulated after SA treatment. The expression levels of *OsOTU18* and *OsOTU20* were all upregulated under the infection of the four viruses and SA treatment (Figure 5A and Figure 8). Although the role of SA in rice antiviral immunity has been illustrated, the molecular function remains to be investigated. Several studies revealed that host immunity mediated by plant hormones, especially JA, plays an important role in defensing in virus infection [33,57,58]. We found that the expression levels of *OsOTU18* and *OsOTU20* were all upregulated under the infection of the four viruses, while their expression levels were significantly downregulated after JA treatment, indicating that they may be susceptible to these four viruses. Studies have shown that BL treatment increases the susceptibility of rice to RBSDV, and JA-mediated defense can inhibit BR-mediated susceptibility to RBSDV infection [53]. We found that the expression levels of *OsOTU18* and *OsOTU20* were all upregulated under BL treatment. However, within *OsOTU1-17* and *OsOTU19*, except for *OsOTU10*, *OsOTU11*, and *OsOTU14*, they all significantly increased after RSV and RB treatment. However, the expression levels of *OsOTU7*, *OsOTU12*, and *OsOTU17* were significantly downregulated after SA and JA treatments. It shows that *OsOTU7, OsOTU12*, and *OsOTU17* were downregulated during RSV and RBSDV infection. The expression levels of *OsOTU1-14* and *OsOTU17* were significantly downregulated after SRBSDV infection. However, after JA treatment, except for *OsOTU7*, *OsOTU11*, *OsOTU12*, and *OsOTU17*, the expression levels of the other *OTU* genes were all upregulated. The results revealed that *OsOTU1-10*, except for *OsOTU7*, may participate in SRBSDV infection. Similarly, we conclude that *OsOTU1*, *OsOTU6*, *OsOTU10*, *OsOTU18*, and *OsOTU20* were upregulated to RSMV infection, while *OsOTU3*, *OsOTU7*, *OsOTU11*, *OsOTU14*, *OsOTU15*, and *OsOTU19* were downregulated. Based on the expression patterns of *OsOTU* family genes under virus and hormone treatments, we speculated that some *OsOTU* family genes are related to plant antiviral defense responses, and there are differences among different rice varieties. To further clarify the suppose, we performed differential expression analysis of resistant ZH5 and susceptible ZS97 following RBSDV infection. In this study, by analysis of the differentially expressed genes between ZH5 and ZS97, we found *OsOTU15* and *OsOTU16* genes displayed a high expression level in the ZH5 plant (Figure 10B). The results suggest that *OsOTU15* and *OsOTU16* may be involved in the antiviral defense response. However, in the follow-up, the transgenic rice of *OsOTU15* and *OsOTU16* will be further constructed, and then inoculated RBSDV with the transgenic rice, which will be further confirmed by phenotype, but it will take a long time. Currently, the lack of understanding of underlying virus resistance gene function has blocked the molecular breeding strategies. The development of resistant cultivars are regarded as an effective approach for virus disease control. Thus, we think that these two genes will be considered as candidate genes of virus-resistant for further analysis.

## Figures and Tables

**Table 1:** The information of the *OTU* gene family in *O. sativa*.

Number	Gene Name	Gene ID Number	Amino Acid Residues	OTU Domain	Peptidase C65 Domain	PI
1	*OsOTU1*	>LOC_Os08g42540.1	274		22–273	4.76
2	*OsOTU2*	>LOC_Os04g33780.1	550	255–366		5.03
3	*OsOTU3*	>LOC_Os04g57480.1	404	51–169		6.69
4	*OsOTU4*	>LOC_Os04g32970.1	228	93–208		8.93
5	*OsOTU5*	>LOC_Os02g07210.2	224	91–206		8.28
6	*OsOTU6*	>LOC_Os06g45850.1	283	150–267		9.14
7	*OsOTU7*	>LOC_Os02g57410.2	300	156–270		5.67
8	*OsOTU8*	>LOC_Os03g64219.1	302	168–282		5.11
9	*OsOTU9*	>LOC_Os04g52850.1	323	180–317	132–312	5.89
10	*OsOTU10*	>LOC_Os02g06890.1	208	11–123		5.08
11	*OsOTU11*	>LOC_Os01g67490.1	360	202–330		10.68
12	*OsOTU12*	>LOC_Os08g39560.1	325	169–297		8.60
13	*OsOTU13*	>LOC_Os09g31280.1	306	166–292		9.10
14	*OsOTU14*	>LOC_Os03g15930.2	224	76–220		8.71
15	*OsOTU15*	>LOC_Os02g32180.1	492		355–473	5.80
16	*OsOTU16*	>LOC_Os02g32190.1	450		248–427	9.58
17	*OsOTU17*	>LOC_Os02g32280.1	459		129–269 289–406	6.96
18	*OsOTU18*	>LOC_Os02g30974.1	605		5–234	8.13
19	*OsOTU19*	>LOC_Os03g39230.1	475	354–469		8.54
20	*OsOTU20*	>LOC_Os04g55840.1	548		228–547	4.64

## Data Availability

All of the materials and data that were used or generated in this study are described and available in the manuscript and Supplementary Files.

## Supplementary Materials

The following supporting information can be downloaded at: https://www.mdpi.com/article/10.3390/v14020392/s1, Table S1: list of primers used in RT-qPCR to determine relative expression levels of rice *OTU* genes. Table S2: properties of the maize OTU proteins identified, including sequence ID and predicted sequence length.

## Appendix A

Three conserved motifs were identified.

motif1 = YVPMDYKEYCKKMRKSGEWGDHVELQAAADVYGVKITVYTS

motif2 = YGLAEVKVEGDGNCQFRALSDQJYRNPEYHKHVRQQIVKQLKEYREHY

motif3 = IVLLFWGEVHYBSLY
