# Supplementary material for: Genome-Wide Identification and Gene Expression Analysis of the OTU DUB Family in Oryza sativa
Source: Viruses. 2022 Feb 14;14(2):392. doi: 10.3390/v14020392 (PMC8878984; doi:10.3390/v14020392)
Supplement: Supplementary file 1 [file viruses-14-00392-s001.zip › Figure S1.pdf]

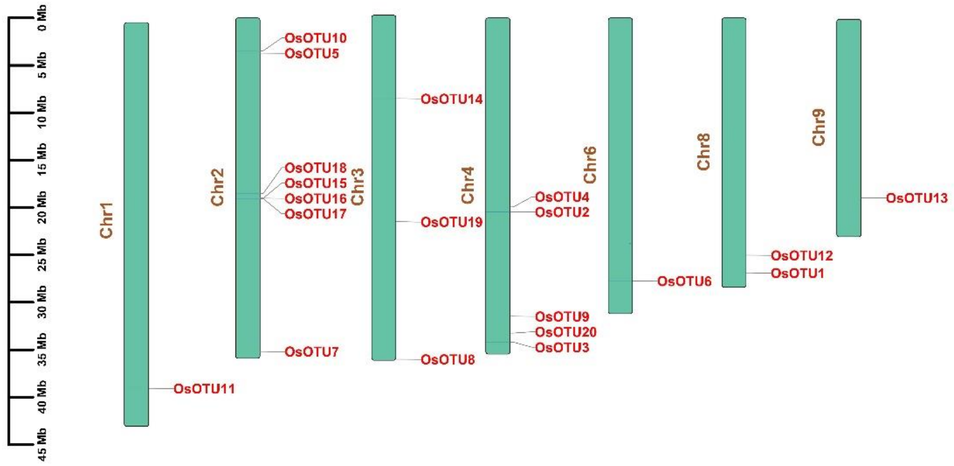

Figure S1. Chromosomal distribution and regional duplication of 20 OTU genes of *Oryza sativa*. The scale bar on the left indicated the length (Mb) of chromosomes.
