# Supplementary material for: Genome-Wide Identification and Gene Expression Analysis of the OTU DUB Family in Oryza sativa
Source: Viruses. 2022 Feb 14;14(2):392. doi: 10.3390/v14020392 (PMC8878984; doi:10.3390/v14020392)
Supplement: Supplementary file 1 [file viruses-14-00392-s001.zip › Table S1-S2.pdf]

Table S1. List of primers used for relative expression level

Gene	Primer	Primer sequence
OsUBQ	RT-UBQ5-2F	ACCACTTCGACCGCCACT
	RT-UBQ5-2R	ACGCCTAAGCCTGCTGGTT
RBSDV-S4	RT-S4-F	ATTTGCGTTTTGCAATTTCC
	RT-S4-R	GCTCTACGACGACCAAATCC
RBSDV-S6	RT-S6-F	AGCGTGTTGAAAACGAGATC
	RT-S6-R	CGCTTTGCAAATTCAACAAG
RBSDV-S10	RT-S10-F	AACAACCGACCAACAATCAC
	RT-S10-R	GAGCAGGAACTTCACGACAG
OsOTU1	RT- OsOTU1-F	AATCCTCCATAGGGGCCGAA
	RT- OsOTU1-R	CCAACGCATCTGATAGGGCA
OsOTU2	RT- OsOTU2-F	GTGCAGTCAGGTATTGGTCC
	RT- OsOTU2-R	TTTGAGCTTCCAGACCCCAT
OsOTU3	RT- OsOTU3-F	TCCATGTCAAGGTCCTGCAA
	RT- OsOTU3-R	ATCCGCATCATCAGAACCCA
OsOTU4	RT- OsOTU4-F	TGAAGCAGCTCAAGGCATGTA
	RT- OsOTU4-R	TCTGTGGTGCCTGATACTGTG
OsOTU5	RT- OsOTU5-F	TTCTATCCCGCACACTCCTC
	RT- OsOTU5-R	CTGCTTCACGACTGACTTCCT
OsOTU6	RT- OsOTU6-F	TCGCCCAAGCAAAGAACAAC
	RT- OsOTU6-R	TGTGATAATCGGGGTTGCGAA
OsOTU7	RT- OsOTU7-F	TTACCCCATGGTGCCAGTTC
	RT- OsOTU7-R	CGTCATATGCCATGGGGACA
OsOTU8	RT- OsOTU8-F	CGAACAAGATGATGCGCCAC
	RT- OsOTU8-R	GCTCCAACCTGTGAAGGAGT
OsOTU9	RT- OsOTU9-F	ATCAGAGGGCAAGGTCGAGT
	RT- OsOTU9-R	ACCGAGTTGTAGTGCTCACC
OsOTU10	RT- OsOTU10-F	TGAAGCGTACTGTGCTTGGA
	RT- OsOTU10-R	GGGCCAATCGACCATTTGTG
OsOTU11	RT- OsOTU11-F	TCTGCCTCATTCCAATCCCC
	RT- OsOTU11-R	TATCGCTTGGCGACTGCTTT
OsOTU12	RT- OsOTU12-F	GACATGCCTTCCCGTGACAA
	RT- OsOTU12-R	TGCATGCCCCATGAATCACA
OsOTU13	RT- OsOTU13-F	ACATGTTTTGCCAGTTCGGG
	RT- OsOTU13-R	GACCCTCAGCATTTGCCTTC
OsOTU14	RT- OsOTU14-F	GGCGTTGGTGAAAGGAATGG
	RT- OsOTU14-R	CCATGGTACTCACGCTCTGG
OsOTU15	RT- OsOTU15-F	ACAAGTTCCACGCATTCCCT
	RT- OsOTU15-R	ATACTCGTCCTTGTGCGAGC
OsOTU16	RT- OsOTU16-F	GGAAAATCGCCTCCTTGCTG
	RT- OsOTU16-R	CAAGCCCAGCTATGTTCGGT
OsOTU17	RT- OsOTU17-F	ATGAGGCAAGCACGTCAAAC
	RT- OsOTU17-R	GAAGGCGATCTTCCTCACCC

OsOTU18	RT- OsOTU18-F	CTCGTGTGTACGCAACCTCT
	RT- OsOTU18-R	GCGGTCCAGATGCTTTTGTT
OsOTU19	RT- OsOTU19-F	GCCAGCACTAACAAAGGGGA
	RT- OsOTU19-R	TCTGCTCGCTGCCTCAAAAA
OsOTU20	RT- OsOTU20-F	GAAGAGGGCACGATGTTGGG
	RT- OsOTU20-R	GGTCGTTGATGGTCCTGTCT

Table S2. The information of the *OTU* gene family in *Zea mays*

number	Gene ID number	Amino acid residues	OTU domain	PI
1	> Zm00014a_002048	167	93-165	9.20
2	> Zm00014a_014232	227	94-208	7.71
3	> Zm00014a_034471	350	214-327	5.29
4	> Zm00014a_008936	300	159-275	5.66
5	> Zm00014a_002135	325	177-316	6.25
6	> Zm00014a_043358	355	159-277	9.00
7	> Zm00014a_019250	253	105-249	9.25
8	> Zm00014a_038080	309	168-295	8.81
9	> Zm00014a_021521	539	243-356	5.22
10	> Zm00014a_025365	310	169-297	8.59
11	> Zm00014a_043850	422	83-210	6.74
12	> Zm00014a_001469	536	245-357	5.37
13	> Zm00014a_015340	156	11-131	6.96
14	> Zm00001d_042481	159	15-143	8.79
15	> Zm00014a_044621	224	94-204	8.97
